# Comparative Analysis of Fatty Acid Bioaccessibility in Commercial Marine Oil Supplements: An In Vitro Integrated Analytical Study

**DOI:** 10.3390/foods13244177

**Published:** 2024-12-23

**Authors:** Thomas Montebugnoli, Giorgia Antonelli, Elena Babini, Ester Maria Vasini, Francesca Danesi, Sigrún Huld Jónasdóttir, María Gudjónsdóttir, Francesco Capozzi, Alessandra Bordoni

**Affiliations:** 1 Department of Agricultural and Food Sciences (DISTAL), University of Bologna, 47521 Cesena, Italy; thomas.montebugnoli2@unibo.it (T.M.); giorgia.antonelli4@unibo.it (G.A.); elena.babini2@unibo.it (E.B.); estermaria.vasini2@unibo.it (E.M.V.); francesca.danesi@unibo.it (F.D.); alessandra.bordoni@unibo.it (A.B.); 2 Interdepartmental Centre for Agri-Food Industrial Research (CIRI Agrifood), University of Bologna, 47521 Cesena, Italy; 3 Consorzio Interuniversitario Risonanze Magnetiche di Metallo Proteine CIRMMP, 50019 Sesto Fiorentino, Italy; 4 DTU Aqua, Technical University of Denmark, 2800 Kgs. Lyngby, Denmark; sjo@aqua.dtu.dk; 5 Faculty of Food Science and Nutrition, University of Iceland, 102 Reykjavík, Iceland; mariagu@hi.is; 6 Matís Food and Biotech R&D, 113 Reykjavík, Iceland

**Keywords:** bioaccessibility, supplements, n-3 LC-PUFAs, in vitro digestion, sustainable source

## Abstract

Zooplankton such as copepods and krill are currently used to produce marine oil supplements, with the aim of helping consumers achieve the recommended intake of n-3 long chain polyunsaturated fatty acids (n-3 LC-PUFAs). Oils from lower trophic levels differ from fish oil in the distribution of lipids into different classes, and this can influence the bioaccessibility of fatty acids, i.e., the percentage of fatty acids that are released into the intestine in a form that can be absorbed by enterocytes. We evaluated fatty acid release after in vitro digestion in four commercial marine oil supplements containing fish, krill and *Calanus finmarchicus* oils using two different analytical approaches, TLC-FID and ^1^H-NMR spectroscopy. The results clearly indicated that the release of free fatty acids (FFAs) after simulated digestion mainly depends on the oil source and is mainly related to the partitioning of lipids into different classes. In fact, the lowest FFA release was detected in Calanus oils, which contain high amounts of wax esters. The different release of FFAs, which appeared secondarily related to encapsulation, can modulate the absorption and blood concentration of the administered n-3 LC-PUFAs and therefore their efficacy. This may partly explain the inconsistencies in intervention studies using marine oil supplements.

## 1. Introduction

It is widely accepted that the omega-3 (n-3) long-chain polyunsaturated fatty acids (n-3 LC-PUFAs) eicosapentaenoic acid (EPA, 20:5n-3) and docosahexaenoic acid (DHA, 22:6n-3) have health benefits for several human diseases, such as cardiovascular diseases [1,2], multiple sclerosis [3] and neurological disorders [4,5]. Major available sources of EPA and DHA include seafoods, and the consumption of fatty fishes twice per week is sufficient to provide 250–300 mg/day of EPA and DHA [6], which corresponds to the recommended daily intake [7]. Although the consumption of these n-3 LC-PUFAs is strongly advised, current daily dietary intakes of EPA and DHA are below the recommended levels in Europe [8], probably due to dietary preferences, geographic reasons, economic status and other reasons [9]. Dietary supplements could overcome low seafood consumption and help consumers to reach the recommended amount of n-3 LC-PUFAs. Various fish oil (FO) supplements like cod liver oil, whole fish body oils, etc., are available on the market to meet such needs. Due to the restrictions on conventional fisheries, the annual production of FO is capped at approximately 1 million metric tons [10], of which about 75% is used by the aquaculture industry to make fish feed [11]. Therefore, there is a need for new and sustainable sources of marine lipids, as well as an improvement in the optimal use of the raw material that is already available.

Some companies have started to harvest lower in the marine food web, also called “fishing down the food web”. Zooplankton, such as copepods and krill, are major primary consumers of plankton in the marine environment [12,13,14] and are currently being utilized for the production of marine oil supplements. The copepod *Calanus finmarchicus* is present in large quantities in the North Atlantic [15] and has a lipid-rich overwintering stage [16]. While the oil extracted from *C. finmarchicus* has been presented as an alternative to marine fishes because of the presence of high concentration of n-3 LC-PUFAs, this practice is highly controversial and not considered sustainable for the marine ecosystem, as these organisms are of great importance as food for commercial fish [17,18,19] and are highly important organisms in ecosystem functions such as promoting biological carbon flux and carbon sequestration [16,20]. Recent research has, however, investigated the potential of utilizing zooplankton as a by-product from pelagic fishing [21,22]. Therefore, it is of high importance to evaluate the bioaccessibility of lipids and fatty acids (FAs) in zooplankton oils to add this nutrition- and health-related information to the sustainability evaluation in the ever-increasing demand for harvesting this resource.

Oils from lower trophic levels often differ from traditional FO in the distribution of lipids into different classes. In first-generation FO supplements, EPA and DHA are bound to a glycerol backbone forming triacylglycerols (TAGs), while in second-generation FO supplements, EPA and DHA are either in the form of ethyl esters or are re-esterified TAGs. Krill oil (KO) has a high content of phospholipids (PLs) in addition to TAGs [23], and in *C. finmarchicus* oil (CO), most of the FAs esterify with long-chain fatty alcohols, forming the lipid class known as wax esters (WEs) [24].

The bioaccessibility of FAs—i.e., the percentage of FAs that are released in the intestine in a form that can be absorbed by enterocytes—depends on the enzymes required to hydrolyze complex lipids in the oils [25,26]; therefore, the distribution of FAs in different lipid classes can influence it. Particularly, in mammalians, WE hydrolysis is assumed to be a slow process because these lipids are poor substrates for lipolytic enzymes, especially pancreatic lipase [27].

The evaluation of the release of FAs in supplements is not trivial, as it modulates bioavailability, i.e., the quantity of FAs that can be absorbed and exert an effect on the human body. Supplements containing similar amounts of LC-PUFAs may therefore have different efficacy due to different bioaccessibility.

In addition, n-3 LC-PUFA supplements are commonly found in capsules, usually made from a soft layer of gelatin. Many manufacturers also use an enteric coating that helps keep the capsule from dissolving until it reaches the small intestine. However, how encapsulation modulates the release of FAs in supplements has not yet been fully clarified [28].

The aim of the present study was to evaluate whether and to what extent the release of FAs from marine oils during digestion is modulated by the form in which they are esterified and by the encapsulation of the oils. To this end, four different commercial n-3 LC-PUFA supplements containing FO, KO or CO were considered, and two different analytical approaches to quantify the different lipid classes before and after in vitro digestion were used. The first one was based on thin-layer chromatography (TLC) with a flame ionization detector (FID); the second one was based on ^1^H nuclear magnetic resonance (^1^H-NMR) spectroscopy. Since, from a chemical point of view, digestion consists of a series of transformations which, at the lipid level, involve the hydrolysis of esters of various nature, ^1^H-NMR spectroscopy is particularly effective. In a single acquisition, the spectrum allows for a complete overview of all the molecules dissolved in the sample, provided that they contain at least one hydrogen atom [29]. As a further advantage, preliminary separation/purification steps are not required, and standards are not needed for quantification. In fact, every signal, regardless of the type of molecule to which the hydrogen atom belongs, has an area proportional to the concentration of the molecule, depending only on the number of equivalent hydrogen atoms corresponding to the signal (e.g., three hydrogen atoms for a methyl group signal and two hydrogen atoms for a methylene group). The peculiarity of NMR spectroscopy is that each hydrogen atom resonates at its specific frequency in the spectrum, defined as a chemical shift, and is expressed as ppm of the carrier frequency. This frequency depends on the chemical environment. For instance, the hydrogen atoms in α of a FA chain resonate at different frequencies depending on whether the FA is in the free (2.378–2.369 ppm) or esterified (2.319–2.250 ppm) form. Moreover, the presence of an adjacent double bond to the same atom group (i.e., DHA) causes an additional shift in their frequencies at higher values (2.400–2.378 ppm for the esterified form or 2.420–2.400 ppm for the free acid) [30]. Changes of a similar extent occur on the alcoholic moieties of esters and glycerides, allowing for the direct observation of chemical transformations such as ester hydrolysis.

## 2. Materials and Methods

### 2.1. Samples

One commercial FO supplement, one commercial Antarctic KO supplement and two commercial CO supplements from different manufacturers (CO-1 and CO-2) were considered. The characteristics of the supplements reported by the manufacturers are listed in Table 1.

### 2.2. In Vitro Digestion

In vitro digestion was performed using the method developed by the European COST action INFOGEST [31]. According to the protocol developed for lipid-rich samples, 0.17 mM lecithin was added to the simulated gastric juice. For each supplement, digestion was performed in duplicate on 5 g of pure oil or 5 g of encapsulated oil.

At the end of duodenal digestion, enzyme activity was stopped by drop-wise HCl addition to pH 3; then, samples were centrifuged at 4500 rpm for 5 min at 4 °C to obtain a soluble fraction (SF) and a pellet. The SF containing the bioaccessible lipids was filtered with a cellulose acetate filter (pore size: 0.2 µm) and stored at −20 °C after adjusting the pH to 7.

### 2.3. Lipid Extraction and Separation by TLC-FID

Total lipids were extracted from not-digested and digested FO, KO and CO according to Bligh and Dyer (1959) [32]. Extraction was performed on 100 mg of not-digested oil or SF obtained after digestion, either without or with capsules. After extraction, the lipids were dried under nitrogen flow and quantified by weighing using an analytical balance.

Separation of the different lipid classes was performed using an IATROSCAN MK-5 TLC-FID. (SES GmbH—Analytical Systems, Bechenheim Germany). The total lipid sample was resolved in chloroform/methanol at a ratio of 2:1, and lipids in chloroform were drawn off and filtered through a phase-separating filter paper (grade 920) and collected in 15 mL vials. The chloroform was left to evaporate under a stream of nitrogen gas. A sub-sample of the lipid was weighted into pre-weighted vials to make a concentration of 15–20 mg oil mL^−1^ hexane. Individual lipid classes were separated by thin-layer chromatography (TLC). A lipid sample was spotted onto silica-coated quartz rods (chromarods) using an automated sample spotter (Model SES 3200/IS-01; both from SES GmbH—Analytical Systems, Bechenheim Germany). Two samples of each oil were analyzed, each in triplicate, to test the instrument’s reproducibility. The lipid classes were separated on the silica rods by development in two solvent systems: hexane/diethyl ether/formic acid (81.5:17.5:1, by volume) followed by hexane/diethyl ether (96:4 by volume). The rods were humidified for 10 min in a constant humidity chamber before developments. The Iatroscan was set to have 20 L min^−1^ air flow, 160 mL min^−1^ hydrogen flow and a scan rate of 30 s. The peaks obtained for each sample were quantified using SES-i-ChromStar 6.4 software (SES GmbH—Analytical Systems, Bechenheim Germany). The peaks were identified by comparison to the retention time of known standards of wax esters (from the copepod *C. finmarchicus*), triacylglycerol (tripalmitin) and cholesterol.

### 2.4. Sample Preparation for Spectroscopic Analysis by ^1^H-NMR

Six drops of the oils were collected from a capsule, pierced with a needle into a microtube and weighted to determine their actual mass (about 200 mg). After the addition of deuterochloroform (CDCl_3_, 500 µL), the solution was mixed vigorously and centrifuged at 14,000 rpm for 5 min at 4 °C. The supernatant was diluted again with CDCl_3_ (1:4 by volume) and the centrifugation step was repeated. Part of the solution (700 µL) was transferred into a 5 mm diameter NMR tube and stored at 4 °C in the autosampler until analysis. For each oil, 3 samples were prepared from different capsules to test the reproducibility.

The SF of the digested oils was weighed, and the pH was adjusted to 2.5–3.0 by adding HCl to convert all the organic acids into their neutral form; this allowed for the partitioning of the molecules released by digestion in favor of chloroform to ensure their complete extraction from the aqueous SF, with the exception of glycerol, short-chain alcohols and glycerophosphocholine, which remain in the aqueous phase. Then, 350 µL of CDCL_3_ were added to the sample and the mixture was mixed vigorously and centrifuged at 14,000 rpm for 5 min at 4 °C. The chloroform phase (bottom) was transferred into an NMR tube, and 350 µL of CDCl_3_ were added to the tube to reach a final volume of 700 µL. For each digested sample, 3 replicates were analyzed.

All samples were analyzed using an AV600 Ultrashield™ Plus AVANCE III™ Spectrometer (Bruker, Karlsruhe, Germany) operating at 600.13 MHz.

The acquisition parameters for analysis were as follows: 1D zg without pre-saturation sequence; correct solvent CDCl_3_; number of time-domain data points TD = 64 k; spectral width SW = 25 ppm; number of scans NS = 8, TD0 = 32 (for a total of 32 × 8 = 256 scans); dummy scans DS = 4; acquisition time AQ = 2.18 s; relaxation delay RD = 8.0 s. The total acquisition time was about 45 min for each sample.

All NMR spectra were manually phased and baseline-corrected using Topspin 3.2 (Bruker Biospin, Rheinstetten, Germany).

### 2.5. Lipid Quantification by ^1^H-NMR

The quantitative determination of the molecules in the samples was performed by integrating specific signals belonging to selected hydrogen atoms of each identified molecule, opportunely free from interferences. The assignment of the signals to the corresponding protons of the different lipid classes (Table 2) was made based on data from the literature for high-resolution ^1^H-NMR spectra of oils from different sources analyzed in CDCl_3_ solvent [33,34,35,36,37,38].

The results of the quantitative evaluation are reported as the molar fraction distribution of the different species and as the ponderal composition (%, *w*/*w*) of the different molecules, assuming an average molar mass of 300 g/mol for the FA chains.

### 2.6. Statistical Analysis

To facilitate comparison between not-digested and digested oils, in the SF of digested oil, the content of the different lipid classes was normalized and expressed in mg/100 mg of the corresponding not-digested oil.

Statistical analysis was performed via one-way analysis of variance using Tukey’s test as post-test. *p* values lower than 0.05 were considered significant.

## 3. Results

The initial stage of fat digestion occurs in the stomach, where gastric lipase begins the hydrolysis of TAGs into diglycerides (DAGs) and free fatty acids (FFAs). Gastric lipase hydrolyses TAGs mainly at position sn-3, containing short- and medium-chain FAs (<C12) that are directly transferred to the blood [39]. However, most fat digestion takes place in the small intestine via pancreatic enzymes and bile salts. The emulsification of fat by bile acids facilitates enzymatic action to further break down fats into FFAs and monoglycerides (MAGs) [40]. After digestion, the hydrolyzed lipid-soluble components (long-chain FAs, 2-MAGs, lysophospholipids, free cholesterol) integrate with bile salts into mixed micelles to diffuse between the intestinal microvilli to interact with the luminal surface of enterocytes [41].

The hydrolysis of more complex lipids into FFAs and MAGs represents a crucial step. Indeed, since the absorption of FAs and MAGs is highly efficient [42], it is presumably the extent of their formation from more complex lipids, as well as the extent of lipid release from the food matrix, that plays a major role in determining the extent of absorption. It is therefore of fundamental importance to evaluate the quantities of FFAs and MAGs that are formed after digestion, particularly in foods or supplements that are consumed because they are sources of specific FAs.

In this work, using two analytical techniques—TLC-FID and ^1^H-NMR spectroscopy—we first evaluated the partitioning of lipids in the different classes in not-digested n-3 LC-PUFA supplements. We then verified the formation of FFAs and MAGs after in vitro digestion.

As shown in Table 3, TLC-FID analysis evidenced that TAGs were more abundant in FO than in other oils, and the concentration of wax esters (WEs) was much higher in CO than in FO and KO, as already reported by Cholewski et al. (2018) [43] and Pedersen et al. (2014) [44], respectively. In KO, which is widely reported to be rich in phospholipids (PLs) [45], TLC-FID analysis evidenced acetone-mobile polar lipids (AMPLs) as the most represented lipid class. Antarctic krill may accumulate large amounts of glycerophospholipids that, together with triacylglycerols, occur as depot lipids [46], and polar lipids, i.e., AMPLs—comprising glycerophospholipids—and PLs are the most represented lipids in microalgae [47], which are consumed by krill [48]. In all not-digested samples, TLC-FID detected small amounts of FFAs.

**Figure 1:**
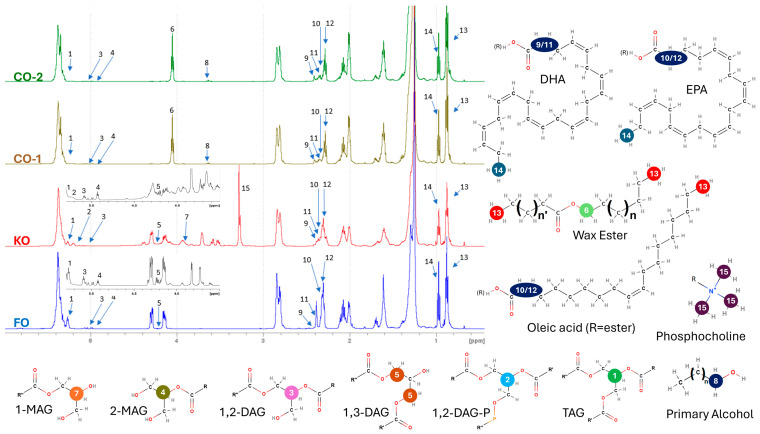
^1^H-NMR spectra (between 0.40 and 6.00 ppm) of not-digested fish oil (FO), krill oil (KO) and Calanus oil (CO-1 and CO-2) supplements in CDCl_3_ solvent. Signal assignment is provided by reporting the label number placed on the spectra also on the corresponding chemical group indicated in the molecular structural formulas. The FO and KO spectra have inserts showing the glyceride region after digestion to better appreciate the signals of the di- and mono-glycerides that are formed during hydrolysis. The extremes of the integration areas are reported in Table 2.

The complete ^1^H-NMR spectra from 0 to 6 ppm of the four not-digested oils are reported in Figure 1, and some spectral regions (0.80–1.40 ppm; 1.40–2.50 ppm; 2.50–3.50 ppm; 3.50–4.50 ppm; 4.50–6.00 ppm; 6.00–10.00 ppm) are enlarged in Figure S1 (Supplementary material) to outline the most significant differences among them.

^1^H-NMR analysis (Table 4) confirmed the TLC-FID results. It is important to clarify that TLC-FID is a chromatographic separation technique that provides an estimate of the weight of different fractions compared to that of three standard compounds mentioned in the Materials and Methods section. This does not allow for a fine subdivision of the chromatographic classes in the different molecular species. NMR, on the other hand, produces precise and accurate information on the molar concentration of the molecular classes present in the mixture, without fractionation, each characterized by a specific chemical functional group. Therefore, the two techniques are not comparable, but it is possible to check the compatibility between the overall data obtained as relative differential quantities before and after digestion. The ^1^H-NMR spectrum of the FO (Figure 1) presented the typical signals of the protons of the glyceryl backbone of TAGs (signal from 5.286 to 5.233 ppm, referring to the hydrogen bound to the glycerol C2), confirming TAGs as the main component. The ^1^H-NMR spectra of CO-1 and CO-2 (Figure 1) significantly differed from the FO spectrum, mainly due to the presence of the signals referring to WEs (primary hydrogens of esterified fatty alcohols, from 4.070 to 4.020 ppm [38]), the latter indicating an even higher proportion due to a separation step which, on the contrary, does not affect the NMR results. The KO spectra (Figure 1) showed a more complex distribution of signals than FO and CO, highlighting a more complex variety of lipid classes. In addition to TAG, the presence of PLs was recognizable by the signals of the glycerophospholipid backbone (5.230–5.170 ppm) and of the choline head (3.330–3.220 ppm) of phosphatidylcholine, the most abundant PLs in krill [49]. In all samples, the amount of FFAs detected by ^1^H-NMR was higher than that detected by TLC-FID. However, the NMR data are particularly robust because the LC-FAs were quantified by integrating signals from both the ω-methyl and β-methylene groups of the FA molecule. The latter resonates at frequencies completely different from those of any other alkyl hydrogen (around 2.2–2.4 ppm vs. 1.1–1.5 ppm). A ratio of 2:3 in the corresponding signal areas confirms that all methyl hydrogen atoms found in the mixture are balanced by the proper number of FA β-methylene hydrogen atoms.

After digestion, the lipid content in the SF was lower than in corresponding not-digested oil regardless of the presence of the capsule (Table 5). We hypothesize that this was mainly related to the different types and levels of emulsifiers used, which can regulate lipid digestibility, since the oil–water interface plays a critical role in modulating the digestive behavior of lipid droplets [50]. Encapsulation played a role in the KO supplement; in fact, when the digestion was performed with capsules, the quantity of lipids in the SF was significantly lower than during digestion without the capsule.

Due to the activity of hydrolytic enzymes during digestion, lipid partition between classes was different in SF than in not-digested oil.

In FO (Table 6), regardless of the presence of the capsule, TLC-FID evidenced a significant decrease in TAG content and an increase in FFAs, DAGs and AMPLs, the latter also including MAGs [51]. Statistical significance was sometimes obscured by within-group variability, which was greater when digestion was performed with capsules. This was probably due to the different dissolution times of the capsules during digestion, which caused different hydrolytic cleavage by the digestive enzymes.

After digestion, the significant decrease in TAGs and the parallel increase in FFAs were confirmed by ^1^H-NMR (Table 7). DAGs and MAGs also increased after digestion, suggesting the progressive hydrolysis of TAGs. Modifications were more relevant when digestion was performed without the capsule. The ^1^H-NMR spectra of digested FO in comparison to not-digested oil are reported in Figure S2.

Comparing the SF of KO to the not-digested oil (Table 8), the TLC-FID analysis highlighted the disappearance of PLs, presumably related to their hydrolysis by pancreatic phospholipases. The absolute content of AMPLS significantly decreased after digestion, particularly when it was performed with the capsule; however, considering the percentage distribution, this class remained the most represented one. No significant modification in absolute FFA content was observed after in vitro digestion of KO.

After digestion, ^1^H-NMR analysis confirmed the decrease in PL content, indicated by the reduction in total P-choline (Table 9). In addition, it allowed for the verification of the almost complete hydrolysis of TAGs. Although the absolute quantity of FFAs was lower in the SF, due to the lower amount of total lipids recovered in this fraction, FFAs accounted for about 61% of the total lipids.

The presence of primary alcohols in the SF of KO may be associated with the formation of lysophospholipids, which possess a free primary alcoholic function at position 1 of the glycerol moiety. As we were not able to assign the other hydrogen atoms of the glycerol moiety to identified signals, for example, those in position 3 (esterified with phosphatidylcholine) or in position 2 (which remains esterified with a FA), we are forced to assign any hydrogen atoms in position 1 (non-esterified) of the glycerol moiety in lysophospholipids as a generic primary alcohol, as it resonates in the typical alcoholic region anyway. The ^1^H-NMR spectra of digested KO in comparison to the not-digested oil are reported in Figure S3.

Regardless of the presence of the capsule, the TLC-FID analysis showed a lower absolute content of WEs in the SF of CO than in the corresponding not-digested oil (Table 10). This decrease was mainly related to the lower lipid content of the SF compared to the not-digested oils, and only partially to WE hydrolysis. Indeed, in the SF, WEs still represented more than 70% of the total lipids, except in CO-2 when the digestion was performed without capsule. The poor hydrolysis of WEs was confirmed by the low amount of FFAs in the SF. Of note, an increase in FFAs was only observed after the digestion of CO-2 without the capsule, concomitant to the increased hydrolysis of WEs. Currently, it is hard to explain why WE hydrolysis and FFA release were significantly different in the two CO supplements; we are sure that the presence of the capsule had an impact on it.

^1^H-NMR analysis confirmed the absence of any hydrolysis of WEs in the CO-1 supplements (Table 11). The observed decrease in absolute WE content was related to the reduced total lipid content in the SF. The mean percentage contribution of each class to the total lipids in the SF was almost the same as in the not-digested sample, indicating that CO-1 did not undergo any hydrolysis. Conversely, some small modification occurred in CO-2, in which a partial hydrolysis of WEs and TAGs was observed after digestion, coupled to a small increase in the contribution of FFAs to total lipids. The ^1^H-NMR spectra of the digested CO in comparison to the not-digested oil are given in Figures S4 and S5.

TAGs, PLs and cholesterol esters are the predominant dietary lipids [52]. During intestinal digestion, pancreatic triacylglycerol lipase and phospholipase hydrolyze TAGs and PLs, respectively [53]. Other digestive enzymes may hydrolyze TAGs, cholesterol esters (CEs), PLs and galactolipids with reaction rates varying accordingly to the substrates, releasing FFAs together with monoacylglycerols (MAGs), lysophospholipids and free cholesterol [54]. As the reaction rates of lipolytic enzymes vary according to their substrates, the bioaccessibility of fatty acids, including n-3 LC-PUFAs, depends on the lipid classes they are bound to. The rate of lipid hydrolysis in the intestine is not a trivial concern, as most lipid classes are too large to be absorbed into the intestinal epithelial cells [53]. It is therefore clear that the n-3 LC-PUFA concentration in supplements is not a reliable reflection of their possible effectiveness, as it does not consider the chemical form of lipids and consequently the bioaccessibility of FAs.

Although some in vitro studies have been carried out to investigate the digestibility of the oils usually used as n-3 LC-PUFA sources in supplements [55,56,57], the different methodologies used for analyzing it make it difficult to compare the results. In addition, to our knowledge, the modulation of digestibility related to the presence of capsules has never been investigated.

To our knowledge, this is the first study comparing FA bioaccessibility in commercial n-3 LC-PUFA supplements using the validated INFOGEST method [31], which was performed without and with the capsule. We did not include gastric lipase in the digestion protocol since in adults, most of the lipid digestion occurs in the intestine. Although this could be considered a limitation, and we are aware that FA bioaccessibility could be higher in vivo, our aim was to compare different n-3 LC-PUFA supplements to evidence whether they have a different release of FAs.

It is known that porcine pancreatin does not provide a complete replacement of the enzymes and lipolytic activities present in human pancreatic juice, since the activity of phospholipase, galactolipase and cholesterol esterase is lower [58]. Usually, this does not have a significant impact on the overall release of FAs, most of which are esterified into TAGs, but in samples containing high amounts of PL or CE, this may represent a limitation of the in vitro digestion method. However, in our study, PLs almost completely disappeared in the digested KO samples, and if lower cholesterol esterase activity had an impact on FA release in COs, the impact was small, as CEs account for 1.2–3.2% of total lipids [59].

The release of FFAs after digestion was quantified using two analytical techniques, TLC-FID and ^1^H-NMR, which gave consistent results, except for some differences arising from the fact that TLC-FID determines the quantities of classes of molecules, which are grouped according to their behavior during the chromatographic elution (e.g., high mobility in acetone) rather than according to their different molecular structure, as is the case of NMR. Consequently, ^1^H-NMR appeared more specific and suitable for quantifying FFAs while also allowing us to discriminate free DHA from all other FAs.

Results clearly indicate that the release of FFAs after simulated digestion significantly depends on the oil source, and it is related to the partition of lipids in the different classes. The lowest FFA release after digestion was detected in COs, which contain high amounts of WEs. In mammalians, WEs hydrolysis is assumed to be a slow process, primarily because they are poor substrates for lipolytic enzymes, especially pancreatic lipase [27]. The poor digestibility of WEs is also suggested by the outbreaks of keriorrhea (oily diarrhea) that are associated with the consumption of large portions of WE-rich fish [60]. Although some studies indicate that mammals can digest moderate amounts of WEs [61] and absorb the liberated FAs and alcohols [62], our results confirmed that CO is not a good source of bioaccessible FAs. That, in addition to the ecological and ethical concerns of directly catching the zooplankton species studied, puts a serious question mark on any sustainability and health claims associated with CO products. In this study, the highest concentration of FFAs and free DHA in the SF of digested oils was found in KO, confirming that FAs in the form of PLs have higher bioavailability than fatty acids in the form of TAGs [63].

**Figure 2:**
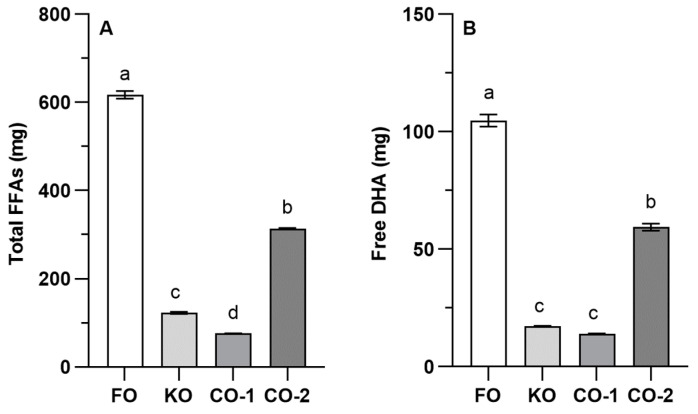
Theoretical amount of (**A**) total FFAs and (**B**) free DHA released after the digestion of the recommended serving size of the different supplements. Free FFAs and DHA were calculated based on serving size as indicated on the product label. Statistical analysis was performed using one-way ANOVA (*p* < 0.001 for both lipid classes) with Tukey’s post-test. Different letters above columns indicate statistical significance at least at *p* < 0.05. FO = fish oil; KO = krill oil; CO = Calanus oil; FFAs = free fatty acids; DHA = docosahexaenoic acid.

In nature, n-3 PUFAs are prevalent as neutral lipids (triglycerides, esters, etc.) and, to a lesser extent, in the form of polar lipids (PL, glycerophospholipids, glycolipids and sphingolipids). The chemical form modulates the release of FAs by digestive enzymes, making them absorbable by enterocytes and increasing their concentration in the blood stream. Therefore, the bioaccessibility of n-3 LC-PUFA is a determining factor of their efficacy. Based on the ^1^H-NMR results reported here, we calculated the amounts of total FFA and free DHA available after the digestion of a recommended serving size of the supplements (Figure 2), and they varied significantly between supplements and were in most cases lower than what was reported on the label (Table 1).

Although the results reported in Figure 2 refer to specific supplements and cannot be generalized, and although they underestimate bioaccessibility since FAs can also be absorbed as MAGs, they highlight an important issue in the use and testing of n-3 LC-PUFA supplements. Indeed, although the evidence suggests that the consumption of foods or supplements containing marine oils may affect chronic diseases and complications of metabolic dysfunctions, the literature on n-3 LC-PUFA supplementation is highly conflicting [64,65], and large trials and meta-analyses have yielded inconsistent findings [1,66,67]. Supplement oxidation is a potential explanation [68], but our results clearly indicate that the source of the marine oil could play a role.

## 4. Conclusions

Although evidence suggests that n-3 LC-PUFAs may affect chronic diseases, the evidence regarding n-3 LC-PUFA supplementation is not as straightforward, and there are some inconsistencies regarding the role of marine oil supplements in both the primary and secondary prevention of cardiovascular diseases. Our results clearly indicate that the source of the marine oil and its encapsulation have a significant impact on the bioaccessibility of fatty acids, thus likely modulating their effectiveness. The potential discrepancy between the theoretical and the actual intake of n-3 LC- PUFAs should be carefully evaluated prior to clinical trials, and our study highlights that in vitro digestion studies coupled with ^1^H-NMR detection represent an effective tool for rapid and cost-effective product screening.

## Figures and Tables

**Table 1:** Characteristics of the commercial supplements reported by the manufacturers.

	FO	KO	CO-1	CO-2
Serving ^1^	3 capsules (1500 mg oil)	2 capsules (1000 mg oil)	1 capsule (500 mg oil)	4 capsules (2100 mg oil)
Total n-3 FA/serving (mg)	505	220	90	442
EPA/serving (mg)	240	120	15	136
DHA/serving (mg)	160	55	20	116
n-3 LC-PUFA source	Fish oil	Antarctic krill (*Euphasia superba*) oil	Calanus (*C. finmarchicus*) oil	Calanus (*C. finmarchicus*) oil
Other ingredients	Gelatin (bovine), glycerol, vitamin E	Gelatin (bovine), glycerol	Gelatin (fish), vegetable glycerin, sorbitol	Gelatin (fish), glycerol, vitamin D3

^1^ When serving was indicated as a range, the maximum value was considered.

**Table 2:** Assignment of the ^1^H-NMR signals of selected lipid classes.

Signal No.	ppm1	ppm2	Assignment	Molecule
1	5.286	5.233	ROCH_a1_H_a2_-C**H_b_**OR’-CH_c1_H_c2_OR’’	TAG
2	5.230	5.170	ROCH_a1_H_a2_-C**H_b_**OR’-CH_c1_H_c2_OPOOR	1,2-DAG-P
3	5.106	5.050	ROCH_a1_H_a2_-C**H_b_**OR’-CH_c1_H_c2_OH	1,2-DAG
4	4.931	4.894	HOCH_a1_H_a2_-C**H_b_**OR-CH_c1_H_c2_OH	2-MAG
5	4.250	4.200	ROC**H_a1_**H_a2_-CH_b_OH-C**H_c1_**H_c2_OR’’	1,3-DAG
6	4.070	4.020	RC**H_2_**O-COR	wax ester
7	3.970	3.877	ROCH_a1_H_a2_-C**H_b_**OH-CH_c1_H_c2_OH	1-MAG
8	3.670	3.620	R-C**H_2_**OH	primary alcohol
9	2.420	2.400	R-C**H_2_**-COOH	DHA free
10	2.378	2.369	R-C**H_2_**-COOH	FFA (no DHA)
11	2.400	2.378	R-C**H_2_**-COOR	DHA bound
12	2.319	2.250	R-C**H_2_**-COOR	BFA (no DHA)
13	0.920	0.840	C**H_3_**-R	all FA except n-3
14	0.995	0.950	C**H_3_**-R	n-3 FA
15	3.330	3.220	R-N(C**H_3_**)_3_	phosphocholine

The extremes of the intervals where signals resonate are indicated as left (ppm1) and right (ppm2) limits. Assignments refer to the hydrogen atom groups (in bold) associated with the signal.

**Table 3:** Partition of lipids in the different classes in not-digested oils, evaluated by TLC-FID.

	FO	KO	CO-1	CO-2	*p*-Value
WEs	4.19 ± 3.84 ^b^	2.98 ± 2.60 ^b^	86.49 ± 1.45 ^a^	78.82 ± 7.13 ^a^	<0.001
TAGs	88.84 ± 3.32 ^a^	8.97 ± 8.66 ^b^	5.10 ± 3.46 ^b^	5.84 ± 6.12 ^b^	<0.001
FFAs	2.00 ± 2.83 ^a^	4.11 ± 3.56 ^a^	3.27 ± 1.04 ^a^	4.01 ± 3.99 ^a^	0.863
ALCs	1.90 ± 2.68 ^a^	1.00 ± 1.72 ^a^	2.73 ± 0.64 ^a^	2.26 ± 2.44 ^a^	0.853
DAGs	1.12 ± 1.58 ^a^	n.d.	n.d.	0.37 ± 0.83 ^a^	0.394
AMPLs	1.59 ± 0.57 ^b^	74.22 ± 7.41 ^a^	2.41 ± 0.34 ^b^	5.76 ± 1.58 ^b^	<0.001
PLs	0.36 ± 0.50 ^a^	8.72 ± 9.45 ^a^	n.d.	2.94 ± 5.65 ^a^	0.411

Data are expressed as m% (*w*/*w*) and are means ± standard deviation (SD) of 2 replicates. Statistical analysis was performed by Student’s *t*-test when comparing two values and by ANOVA (one-way analysis of variance) using Tukey’s test as post-test when comparing three or more values. *p* < 0.05 was considered significant. Different letters in the same row indicate statistical significance. WEs = sterol esters and wax esters; TAGs = triglycerides; FFAs = free fatty acids; ALC = free aliphatic alcohols; DAGs = diglycerides; AMPLs = acetone-mobile polar lipids; PLs = phospholipids and other acetone immobile polar lipids; FO = fish oil; KO = krill oil; CO = Calanus oil; n.d. = not detectable.

**Table 4:** Partition of lipids in the different classes in not-digested oils, evaluated by ^1^H-NMR.

	FO	KO	CO-1	CO-2	*p*-Value
TAGs	68.74 ± 0.16 ^a^	26.24 ± 0.22 ^b^	0.91 ± 0.06 ^c^	1.26 ± 0.06 ^c^	<0.001
1,3-DAGs	1.43 ± 0.01 ^a^	0.94 ± 0.34 ^b^	0.06 ± 0.01 ^c^	0.44 ± 0.03 ^c^	<0.001
1,2-DAGs	2.61 ± 0.02 ^a^	2.97 ± 0.75 ^a^	1.16 ± 0.30 ^b^	0.17 ± 0.004 ^b^	<0.001
1,2-DAG-P	n.d.	5.80 ± 0.15	n.d.	n.d.	
2-MAGs	0.40 ± 0.02 ^a^	0.10 ± 0.04 ^b^	0.02 ± 0.00 ^c^	0.05 ± 0.01 ^b,c^	<0.001
1-MAGs	0.50 ± 0.02 ^c^	2.79 ± 0.21 ^a^	1.18 ± 0.01 ^b^	0.14 ± 0.004 ^d^	<0.001
WEs	0.02 ± 0.00 ^c^	0.38 ± 0.16 ^c^	63.86 ± 0.59 ^b^	70.30 ± 0.03 ^a^	<0.001
Primary alcohol	0.11 ± 0.01 ^c^	0.21 ± 0.02 ^c^	3.04 ± 0.45 ^a^	2.12 ± 0.03 ^b^	<0.001
total P-choline	0.04 ± 0.004 ****	21.83 ± 0.31	n.d.	n.d.	<0.0001
FFAs (except DHA)	21.04 ± 0.14 ^c^	36.09 ± 0.60 ^a^	24.25 ± 0.15 ^b^	19.06 ± 0.08 ^d^	<0.001
Free DHA	5.13 ± 0.05 ^b^	2.67 ± 0.04 ^d^	5.52 ± 0.03 ^a^	3.70 ± 0.06 ^c^	<0.001

Data are expressed as % (*w*/*w*) and are means ± SD of 3 replicates. Statistical analysis was performed by Student’s *t*-test when comparing two values and by ANOVA (one-way analysis of variance) using Tukey’s test as post-test when comparing three or more values. *p* < 0.05 was considered significant. **** means *p* ≤ 0.0001. Different letters in the same row indicate statistical significance. TAGs = triglycerides; DAGs = diglycerides; MAGs = monoglycerides; WEs = wax esters; FFAs = free fatty acids; FO = fish oil; KO = krill oil; CO = Calanus oil. n.d. = not detectable.

**Table 5:** Total lipids recovered in the SF of the different oils after in vitro digestion without or with the capsule.

	FO	KO	CO–1	CO–2	*p*-Value
Without capsule	61.51 ± 1.54 ^a^	44.00 ± 0.54 ^c^	61.99 ± 0.34 ^a^	53.24 ± 2.76 ^b^	0.001
With capsule	63.55 ± 2.90 ^a^	19.21 ± 0.31 ^c^ ***	53.95 ± 4.03 ^a,b^	53.20 ± 0.44 ^b^	<0.001

Data are expressed in mg/100 mg not-digested oil and are means ± SD (n = 2). Statistical analysis was performed by one-way ANOVA using Tukey’s test as post-test, and *p* < 0.05 was considered significant. Different letters in the same row indicate statistical significance. In each oil, differences related to the presence of the capsule during digestion were evaluated using Student’s *t*-test (*** *p* < 0.001). FO = fish oil; KO = krill oil; CO = Calanus oil.

**Table 6:** Partition of lipids in the different classes in SF of FO after digestion with or without capsules, evaluated by TLC-FID.

	Not Digested	SF (Digested w/o Capsule)	SF (Digested with Capsule)	*p*-Value
WEs	4.19 ± 3.84 ^a^	0.85 ± 0.81 ^a^ (1.38%)	3.49 ± 4.93 ^a^ (5.32%)	0.55
TAGs	88.84 ± 3.32 ^a^	15.57 ± 1.49 ^b^ (25.31%)	12.22 ± 1.55 ^b^ (19.20%)	<0.001
FFAs	2.00 ± 2.83 ^a^	10.95 ± 0.90 ^a^ (17.80%)	26.26 ± 19.94 ^a^ (42.08%)	0.25
ALCs	1.90 ± 2.68	n.d.	n.d.	
DAGs	1.12 ± 1.58 ^a^	9.53 ± 1.60 ^a^ (15.49%)	7.13 ± 4.21 ^a^ (11.08%)	0.10
AMPLs	1.59 ± 0.57 ^a^	21.58 ± 3.05 ^a^ (35.08%)	10.08 ± 9.58 ^a^ (15.54%)	0.09
PLs	0.36 ± 0.50 ^a^	3.02 ± 1.50 ^a^ (4.91%)	4.36 ± 2.57 ^a^ (6.78%)	0.19

Data are expressed as mg/100 mg of not-digested oil and are means ± SD of 2 replicates. Statistical analysis was performed by Student’s *t*-test when comparing two values and by one-way ANOVA with Tukey’s test as post-test when comparing three values. *p* < 0.05 was considered significant. Different letters in the same row indicate statistical significance. The mean percentage contribution of each class to total lipids in SF is reported in parentheses. SF = soluble fraction; WEs = sterol esters and wax esters; TAGs = triglycerides; FFAs = free fatty acids; ALCs = free aliphatic alcohols; DAGs = diglycerides; AMPLs = acetone-mobile polar lipids; PLs = phospholipids and other acetone immobile polar lipids; n.d. = not detectable.

**Table 7:** Partition of lipids in the different classes in SF of FO after digestion with or without capsules, evaluated by ^1^H-NMR.

	Not Digested	SF (Digested w/o Capsule)	SF (Digested with Capsule)	*p*-Value
TAGs	68.74 ± 0.16 ^a^	19.02 ± 7.76 ^b^ (19.02%)	15.45 ± 0.05 ^b^ (24.35%)	<0.001
1,3-DAGs	1.43 ± 0.01 ^a^	2.21 ± 0.81 ^a^ (2.2%)	1.07 ± 0.42 ^a^ (1.7%)	0.14
1,2-DAGs	2.61 ± 0.02 ^b^	7.75 ± 2.22 ^a^ (7.75%)	5.41 ± 0.66 ^a,b^ (8.54%)	0.02
1,2-DAGs-P	n.d.	n.d.	n.d.	
2-MAGs	0.40 ± 0.02 ^b^	3.62 ± 1.21 ^a^ (3.62%)	2.31 ± 0.71 ^a,b^ (3.67%)	0.02
1-MAGs	0.50 ± 0.02 ^b^	2.90 ± 1.39 ^a^ (2.09%)	2.13 ± 0.45 ^a,b^ (3.35%)	0.05
WEs	0.02 ± 0.001 **	n.d.	0.01 ± 0.002 (0.02%)	0.0045
Primary alcohol	0.11 ± 0.01 ^a^	0.12 ± 0.08 ^a^ (0.12%)	0.04 ± 0.03 ^a^ (0.07%)	0.24
Total P-choline	0.04 ± 0.005	n.d.	n.d.	
FFAs (except DHA)	21.04 ± 0.14 ^b^	54.27 ± 11.92 ^a^ (54.27%)	30.35 ± 5.38 ^b^ (47.62%)	0.01
Free DHA	5.13 ± 0.05 ^b^	10.10 ± 1.15 ^a^ (10.10%)	6.79 ± 0.28 ^b^ (10.68%)	0.002

Data are expressed as mg/100 mg of not-digested oil and are means ± SD of two biological replicates, except for the not-digested oil, which was analyzed in three biological replicates. Each biological replicate was evaluated in triplicate. Statistical analysis was performed by Student’s *t*-test when comparing two values and by ANOVA (one-way analysis of variance) using Tukey’s test as post-test when comparing three values. *p* < 0.05 was considered significant. ** means *p* ≤ 0.01. Different letters in the same row indicate statistical significance. The mean percentage contribution of each class to total lipids in SF is reported in parentheses. TAGs = triglycerides; DAGs = diglycerides; MAGs = monoglycerides; WEs = wax esters; FFAs = free fatty acids; DHA = docosahexaenoic acid; FO = fish oil. n.d. = not detectable.

**Table 8:** Partition of lipids in the different classes in SF of KO after digestion with or without capsules, evaluated by TLC-FID.

	Not Digested	SF (Digested w/o Capsule)	SF (Digested with Capsule)	*p*-Value
WEs	2.98 ± 2.60	n.d.	0.12 ± 0.16 (0.62%)	0.26
TAGs	8.97 ± 8.66 ^a^	5.89 ± 1.98 ^a^ (13.39%)	1.21 ± 0.58 ^a^ (6.30%)	0.42
FFAs	4.11 ± 3.56 ^a^	2.08 ± 0.65 ^a^ (4.73%)	2.45 ± 0.14 ^a^ (12.75%)	0.63
ALCs	1.00 ± 1.72 ^a^	0.60 ± 0.85 ^a^ (1.36%)	0.31± 0.44 ^a^ (1.61%)	0.84
DAGs	n.d.	0.10 ± 0.14 (0.23%)	0.72 ± 1.01 (3.75%)	0.48
AMPLs	74.22 ± 7.41 ^a^	35.32 ± 2.59 ^b^ (80.27%)	12.44 ± 0.77 ^c^ (64.76%)	0.002
PLs	8.72 ± 9.45	n.d.	n.d.	

Data are expressed as mg/100 mg not-digested oil and are means ± SD of 2 replicates. Statistical analysis was performed by Student’s *t*-test when comparing two values and by one-way ANOVA with Tukey’s test as post-test when comparing three values. *p* < 0.05 was considered significant. Different letters in the same row indicate statistical significance. The mean percentage contribution of each class to total lipids in SF is reported in parentheses. SF = soluble fraction; WEs = sterol esters and wax esters; TAGs = triglycerides; FFAs = free fatty acids; ALC = free aliphatic alcohols; DAG = diglycerides; AMPLs = acetone-mobile polar lipids; PLs = phospholipids and other acetone immobile polar lipids; n.d. = not detectable.

**Table 9:** Partition of lipids in the different classes in SF of KO after digestion with or without capsules, evaluated by ^1^H-NMR.

	Not Digested	SF (Digested w/o Capsule)	SF (Digested with Capsule)	*p*-Value
TAGs	26.24 ± 0.22 ^a^	0.35 ± 0.16 ^b^ (0.80%)	0.48 ± 0.14 ^b^ (2.51%)	<0.001
1,3-DAGs	0.94 ± 0.34 ^a^	0.23 ± 0.06 ^a,b^ (0.52%)	0.01 ± 0.01 ^b^ (0.05%)	0.03
1,2-DAGs	2.97 ± 0.75 ^a^	0.51 ± 0.10 ^b^ (1.15)	0.47 ± 0.12 ^b^ (2.43%)	0.010
1,2-DAGs-P	5.80 ± 0.15 ^a^	0.87 ± 0.08 ^b^ (1.97%)	0.36 ± 0.02 ^c^ (1.89%)	<0.001
2-MAGs	0.10 ± 0.04 ^b^	0.62 ± 0.28 ^a,b^ (1.41%)	0.69 ± 0.17 ^a^ (3.61%)	0.03
1-MAGs	2.79 ± 0.21 ^a^	0.70 ± 0.03 ^b^ (1.59%)	0.35 ± 0.02 ^b^ (1.81%)	<0.001
WEs	0.38 ± 0.16 ^a^	0.05 ± 0.04 ^a^ (0.11%)	0.06 ± 0.08 ^a^ (0.34%)	0.06
Primary alcohol	0.21 ± 0.02 ^a^	3.03 ± 3.35 ^a^ (6.93%)	1.19 ± 1.65 ^a^ (6.28%)	0.35
Total P-choline	21.83 ± 0.31 ^a^	5.72 ± 0.32 ^b^ (12.99%)	2.32 ± 0.20 ^c^ (12.05%)	<0.001
FFAs (except DHA)	36.09 ± 0.60 ^a^	27.07 ± 1.94 ^b^ (61.59%)	11.87 ± 1.88 ^c^ (61.74%)	<0.001
Free DHA	2.67 ± 0.04 ^b^	4.86 ± 1.12 ^a^ (11.03%)	1.40 ± 0.47 ^b^ (7.28%)	0.01

Data are expressed as mg/100 mg of not-digested oil and are means ± SD of two biological replicates, except for the not-digested oil, which was analyzed in three biological replicates. Each biological replicate was evaluated in triplicate. Statistical analysis was performed by one-way ANOVA using Tukey’s test as post-test, and *p* < 0.05 was considered significant. Different letters in the same row indicate statistical significance. The mean percentage contribution of each class to total lipids in SF is reported in parentheses. TAGs = triglycerides; DAGs = diglycerides; MAGs = monoglycerides; WEs = wax esters; FFAs = free fatty acids; DHA = docosahexaenoic acid; KO = krill oil.

**Table 10:** Partition of lipids in the different classes in SF of COs after digestion with or without capsules, evaluated by TLC-FID.

**CO-1**	**Not Digested**	**SF (Digested w/o Capsule)**	**SF (Digested with Capsule)**	***p*-Value**
WEs	86.49 ± 1.45 ^a^	45.76 ± 1.53 ^b^ (73.82%)	41.95 ± 7.03 ^b^ (77.76%)	0.003
TAGs	5.10 ± 3.46 ^a^	2.66 ± 1.55 ^a^ (4.29%)	0.21 ± 0.29 ^a^ (0.39%)	0.23
FFAs	3.27 ± 1.04	n.d.	1.11 ± 1.01 (2.06%)	0.16
ALCs	2.73 ± 0.64	n.d.	0.89 ± 0.40 (1.65%)	0.07
DAGs	n.d.	n.d.	0.29 ± 0.41 (0.54%)	
AMPLs	2.41 ± 0.34 ^a^	12.66 ± 4.04 ^a^ (20.42%)	8.98 ± 4.21 ^a^ (16.65%)	0.12
PLs	n.d.	0.50 ± 0.71 ^b^ (0.81%)	0.59 ± 0.31 ^b^ (1.09%)	0.88
**CO-2**	**Not Digested**	**SF (Digested w/o Capsule)**	**SF (Digested with Capsule)**	***p*-Value**
WEs	78.82 ± 7.13 ^a^	24.05 ± 4.54 ^b^ (45.17%)	44.20 ± 0.96 ^b^ (83.08%)	0.003
TAGs	5.84 ± 6.12	0.66 ± 0.68 (1.24%)	tr	0.36
FFAs	4.01 ± 3.99 ^a^	16.19 ± 8.97 ^a^ (30.41%)	2.25 ± 1.77 ^a^ (4.23%)	0.17
ALCs	2.26 ± 2.44 ^a^	0.26 ± 0.10 ^a^ (0.49%)	0.38 ± 0.53 ^a^ (0.71%)	0.41
DAGs	0.37 ± 0.83	0.65 ± 0.92 (1.22%)	n.d.	0.78
AMPLs	5.76 ± 1.58 ^a^	10.24 ± 3.55 ^a^ (19.23%)	5.71 ± 1.40 ^a^ (10.73%)	0.24
PLs	2.94 ± 5.65	1.29 ± 1.83 (2.42%)	n.d.	0.73

Data are expressed as mg/100 mg not-digested oil and are means ± SD of 2 replicates. Statistical analysis was performed by Student’s *t*-test when comparing two values and by one-way ANOVA with Tukey’s test as post-test when comparing three values. *p* < 0.05 was considered significant. Different letters in the same row indicate statistical significance. The mean percentage contribution of each class to total lipids in SF is reported in parentheses. WEs = sterol esters and wax esters; TAGs = triglycerides; FFAs = free fatty acids; ALC = free aliphatic alcohols; DAG = diglycerides; AMPLs = acetone-mobile polar lipids; PLs = phospholipids and other acetone immobile polar lipids; n.d. = not detectable.

**Table 11:** Partition of lipids in the different classes in SF of COs after digestion with or without capsules, evaluated by ^1^H-NMR.

**CO-1**	**Not Digested**	**SF (Digested w/o Capsule)**	**SF (Digested with Capsule)**	***p*-Value**
TAGs	0.91 ± 0.06 ^a^	0.51 ± 0.09 ^b^ (0.82%)	0.50 ± 0.05 ^b^ (0.92%)	0.004
1,3-DAGs	0.06 ± 0.01 ^a^	0.03 ± 0.004 ^b^ (0.06%)	0.03 ± 0.004 ^b^ (0.05%)	0.02
1,2-DAGs	1.16 ± 0.30 ^a^	1.14 ± 0.60 ^a^ (1.84%)	0.63 ± 0.05 ^a^ (1.17%)	0.34
1,2-DAGs-P	n.d.	n.d.	n.d.	
2-MAGs	<0.01	<0.01	<0.01	
1-MAGs	1.18 ± 0.01 ^a^	0.74 ± 0.03 ^b^ (1.20%)	0.65 ± 0.06 ^b^ (1.20%)	<0.001
WEs	63.86 ± 0.59 ^a^	38.83 ± 0.33 ^b^ (62.64%)	34.61 ± 2.79 ^b^ (64.13%)	<0.001
Primary alcohol	3.04 ± 0.45 ^a^	2.28 ± 0.24 ^a,b^ (3.67%)	1.54 ± 0.03 ^b^ (2.86%)	0.02
Total P-choline	n.d.	n.d.	n.d.	
FFAs (except DHA)	24.25 ± 0.15 ^a^	14.95 ± 0.24 ^b^ (24.12%)	12.99 ± 0.85 ^c^ (24.09%)	<0.001
Free DHA	5.52 ± 0.03 ^a^	3.49 ± 0.08 ^b^ (5.63%)	3.00 ± 0.26 ^b^ (5.56%)	<0.001
**CO-2**	**Not Digested**	**SF (Digested w/o Capsule)**	**SF (Digested with Capsule)**	***p*-Value**
TAGs	1.26 ± 0.06 ^a^	0.25 ± 0.07 ^b^ (0.48%)	0.35 ± 0.02 ^b^ (0.66%)	<0.001
1,3-DAGs	0.44 ± 0.03 ^a^	0.03 ± 0.01 ^b^ (0.05%)	0.03 ± 0.01 ^b^ (0.05%)	<0.001
1,2-DAGs	0.17 ± 0.10 ^a^	0.43 ± 0.21 ^a^ (0.81%)	0.34 ± 0.21 ^a^ (0.63%)	0.25
1,2-DAGs-P	n.d.	n.d.	n.d.	
2-MAGs	0.05 ± 0.01	n.d.	n.d.	
1-MAGs	0.14 ± 0.002 ^b^	0.58 ± 0.07 ^a^ (1.08%)	0.37 ± 0.26 ^a,b^ (0.70%)	0.06
WEs	70.30 ± 0.03 ^a^	34.42 ± 0.59 ^b^ (64.71%)	34.95 ± 0.09 ^b^ (65.70%)	<0.001
Primary alcohol	2.12 ± 0.03 ^a^	1.87 ± 0.26 ^a^ (3.50%)	1.75 ± 0.14 ^a^ (3.29%)	0.11
Total P-choline	n.d.	n.d.	n.d.	
FFAs (except DHA)	19.06 ± 0.08 ^a^	12.84 ± 1.50 ^b^ (23.94%)	12.60 ± 0.69 ^b^ (23.68%)	0.001
Free DHA	6.45 ± 0.10 ^a^	2.82 ± 0.17 ^b^ (5.30%)	2.81 ± 0.03 ^b^ (5.29%)	<0.001

Data are expressed as mg/100 mg of not-digested oil and are means ± SD of two biological replicates, except for the not-digested oil, which was analyzed in three biological replicates. Each biological replicate was evaluated in triplicate. Statistical analysis was by one-way ANOVA using Tukey’s test as post-test, and *p* < 0.05 was considered significant. Different letters in the same row indicate statistical significance. The mean percentage contribution of each class to total lipids in SF is reported in parentheses. TAGs = triglycerides; DAGs = diglycerides; MAGs = monoglycerides; WEs = wax esters; FFAs = free fatty acids; DHA = docosahexaenoic acid; KO = krill oil. n.d. = not detectable.

## Data Availability

The original contributions presented in the study are included in the article and supplementary materials, further inquiries can be directed to the corresponding author.

## Supplementary Materials

The following supporting information can be downloaded at https://www.mdpi.com/article/10.3390/foods13244177/s1: Figure S1: Expansions of specific regions of ^1^H-NMR spectra acquired for different oils from not-digested supplements; Figure S2: Zoom in on the glyceride region in the ^1^H-NMR spectra of not-digested fish oil and digestate of the corresponding supplements, with or without encapsulation; Figure S3: 1H-NMR spectra of not-digested krill oil and digestate of the corresponding supplements with or without encapsulation; Figure S4: ^1^H-NMR spectra of not-digested Calanus oil n.1 and digestate of the corresponding supplements with or without encapsulation; Figure S5: ^1^H-NMR spectra of not-digested Calanus oil n.2 and digestate of the corresponding supplements with or without encapsulation.
