# Supplementary material for: Comparative Analysis of Fatty Acid Bioaccessibility in Commercial Marine Oil Supplements: An In Vitro Integrated Analytical Study
Source: Foods. 2024 Dec 23;13(24):4177. doi: 10.3390/foods13244177 (PMC11675117; doi:10.3390/foods13244177)
Supplement: Supplementary file 1 [file foods-13-04177-s001.zip › foods-3346445-supplementary.pdf]

Supplementary material

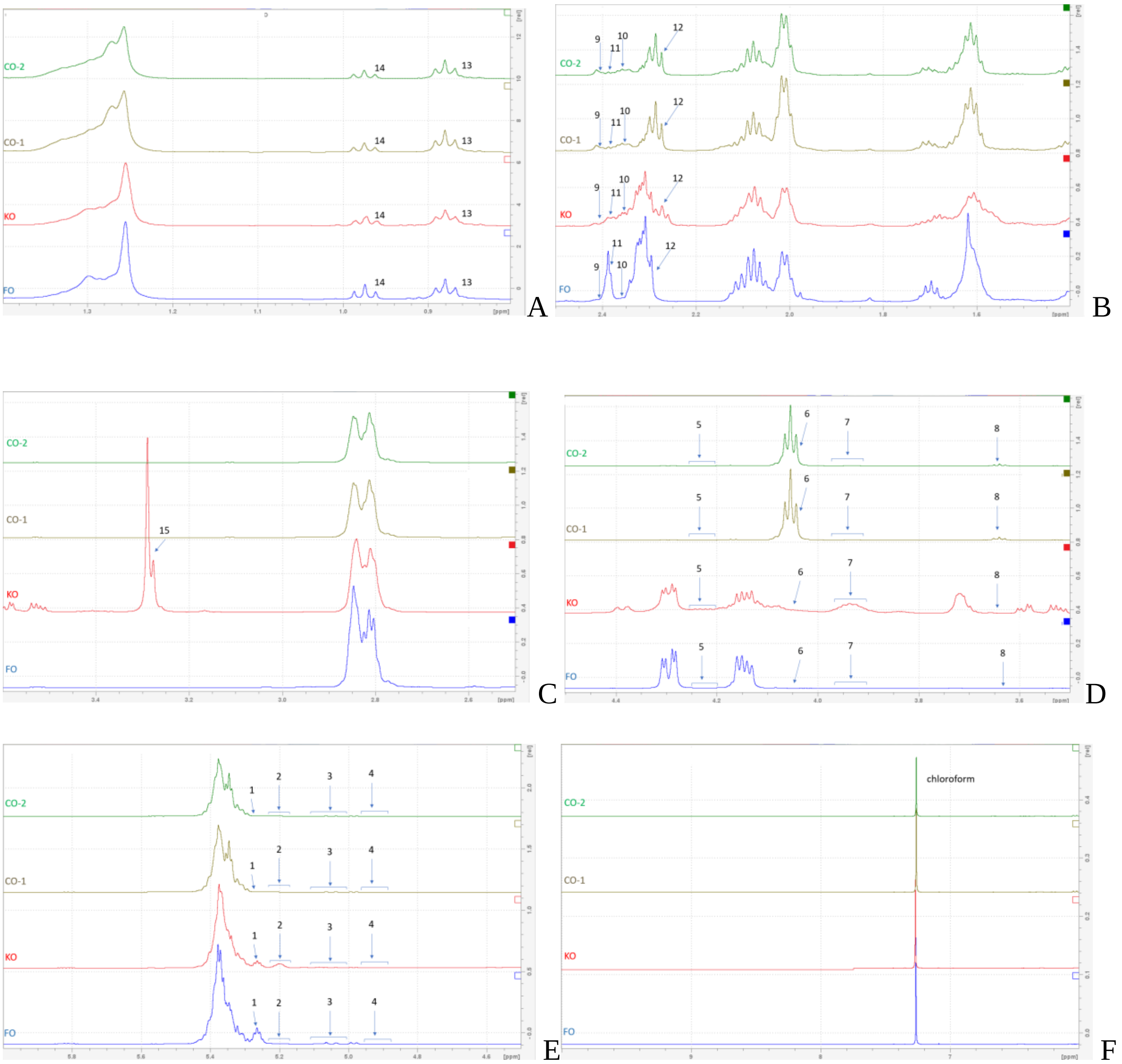

Figure S1. Expansions of specific regions ^{1}H-NMR spectra recorded in $CDCl_3$ solvent on fish oil (FO, in blue), krill oil (KO, in red), *Calanus* oils (CO-1 in brown and CO-2 in green) from not-digested supplements, in the regions of: A (0.84-1.40 ppm) terminal CH_3 of all FAs; B (1.40-2.50 ppm) β-methylene groups of DHA and other FA free and bound; C (2.50-3.50 ppm) phosphocholine signal - visible only in the KO spectrum; D (3.50-4.50 ppm) esterified 1° alcohol (4.05 ppm) typical of CO-1 and CO-2 oil; E (4.50-6.00 ppm) MAG, 1,2-DAG, 1,3-DAG and 1,2-DAG-P; F (6.00-10.00 ppm), the downfield region with the chloroform signal.

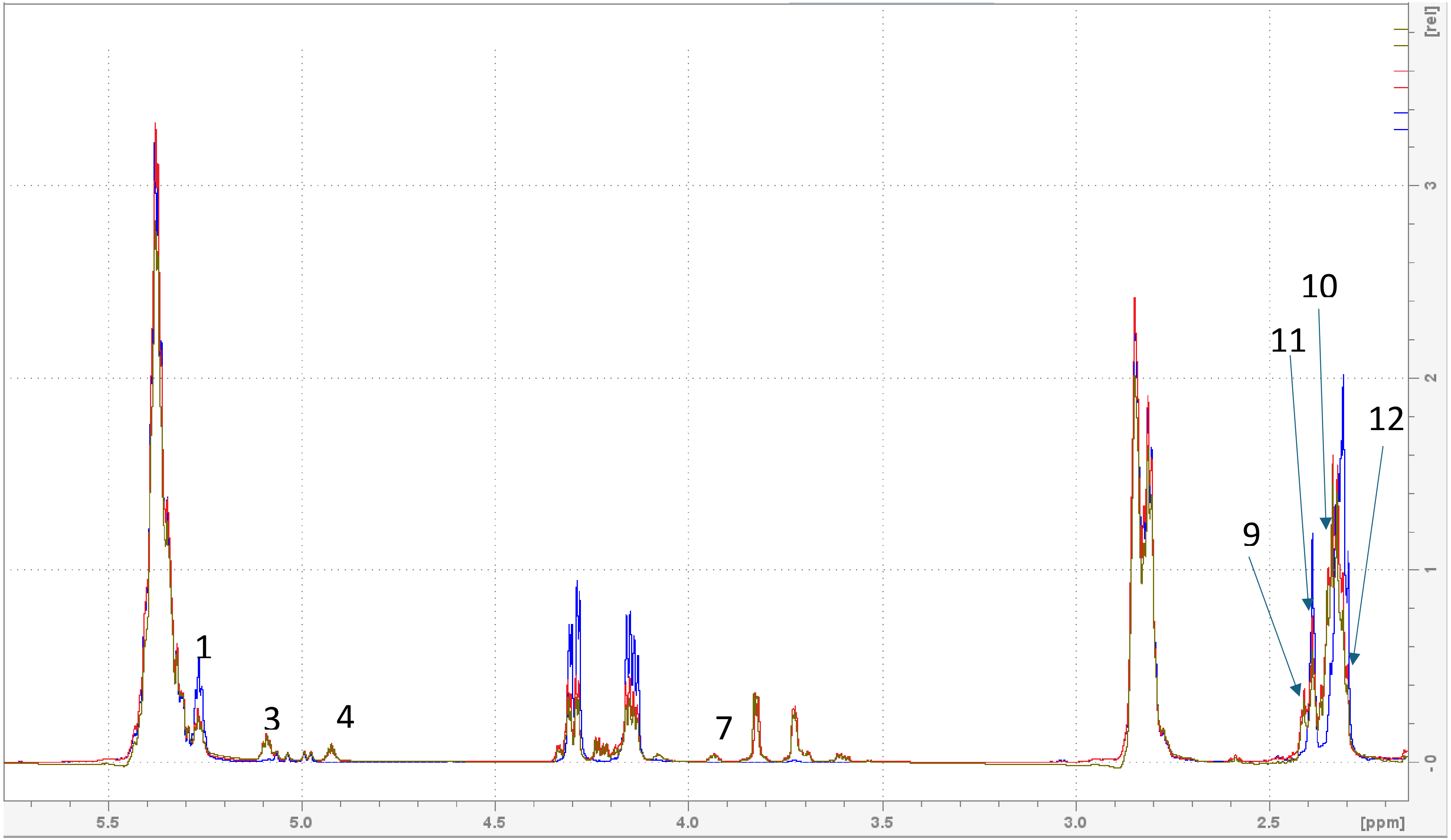

Figure S2. Zoom of the glyceride region (2.20-5.80 ppm) of ^{1}H-NMR spectra recorded on not-digested FO (blue), and on digestate of encapsulated (red) or non-encapsulated (brown) supplements. Peak assignment is provided in Table 2.

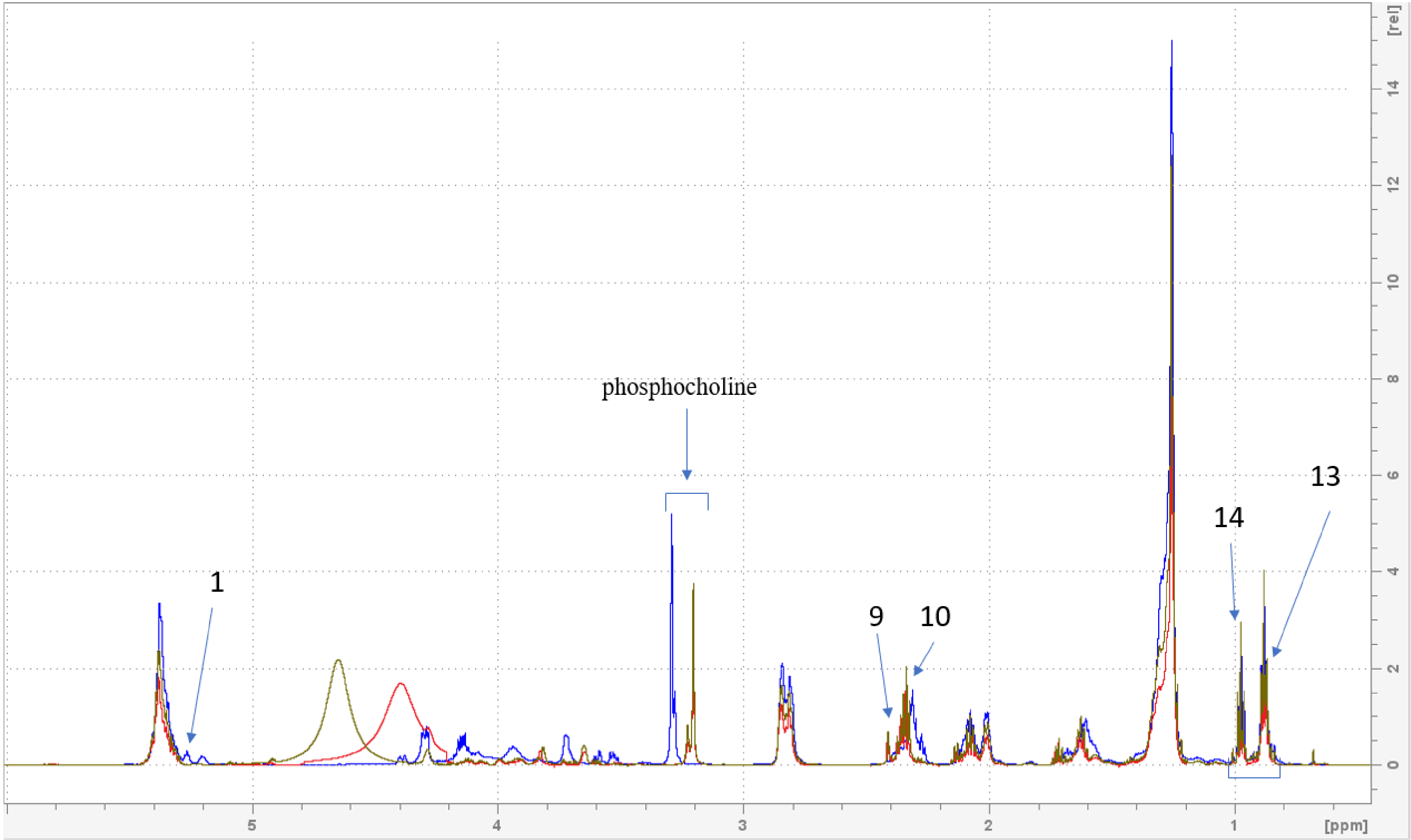

Figure S3. ^{1}H-NMR spectra recorded on not-digested KO (blue), and on digestate of encapsulated (red) or non-encapsulated (brown) KO supplements. Peak assignment is provided in Table 2.

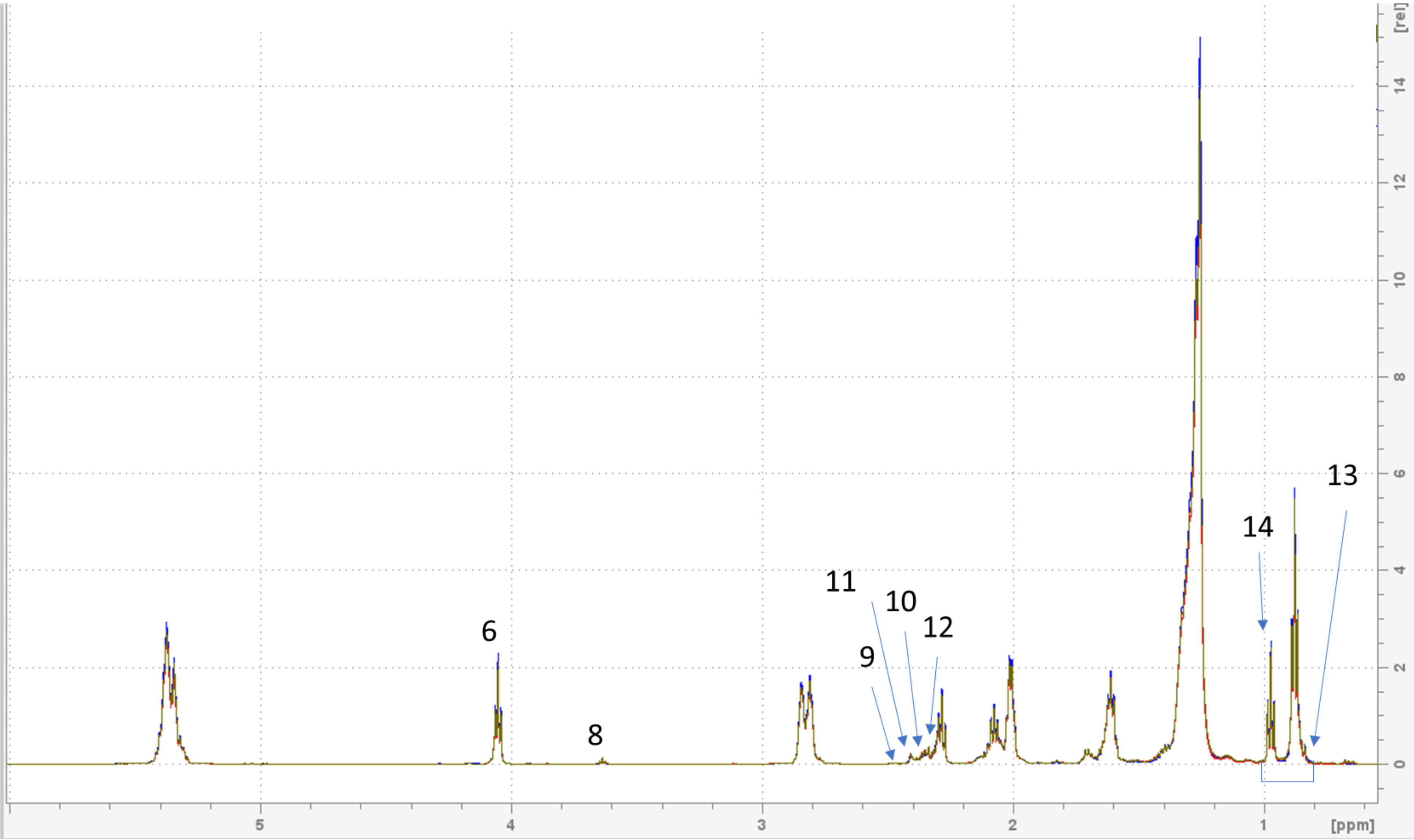

Figure S4. ¹H-NMR spectra recorded on not-digested CO-1 (blue), and on digestate of encapsulated (red) or non-encapsulated (brown) CO-1 supplements. Peak assignment is provided in Table 2.

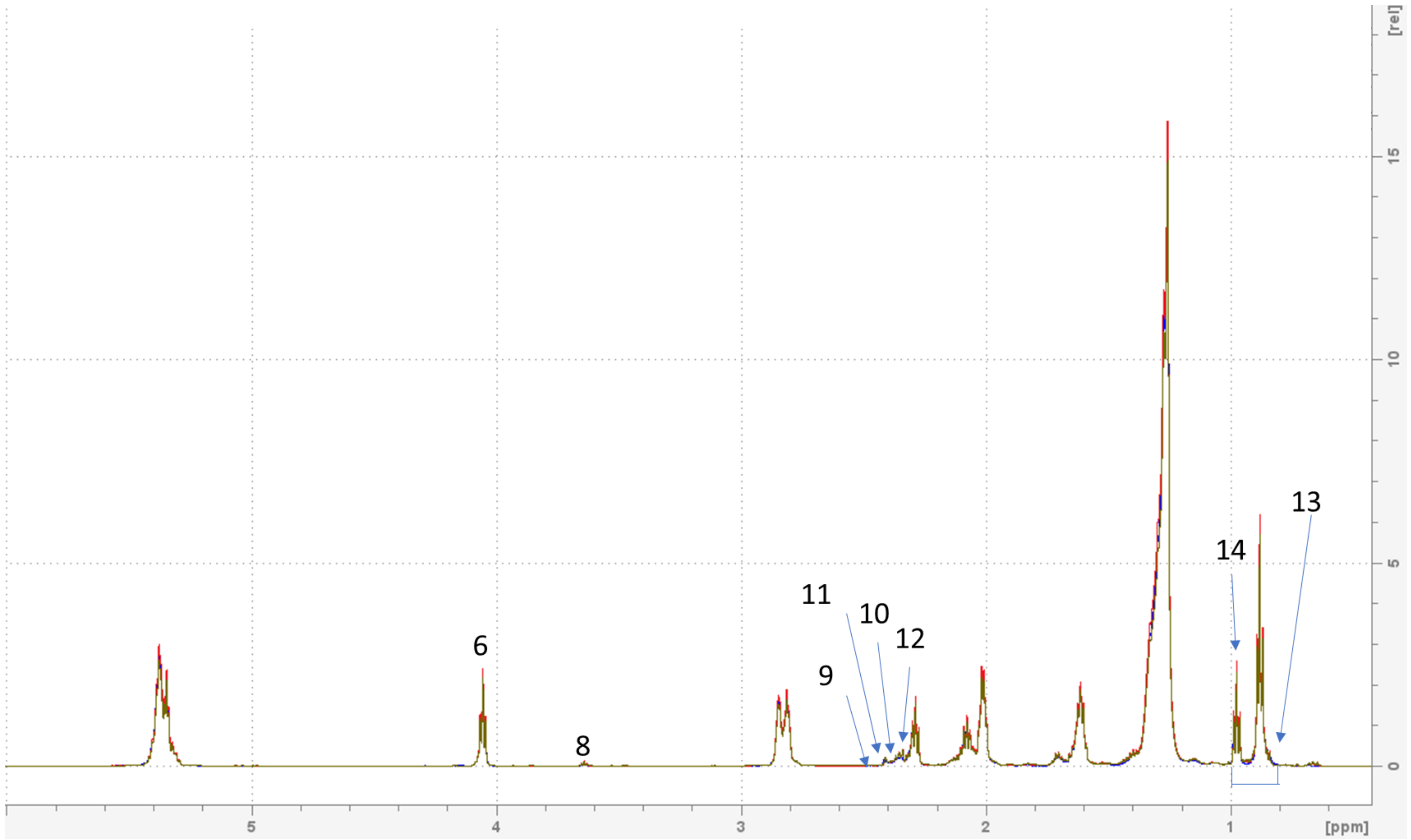

Figure S5. ^{1}H-NMR spectra recorded on undigested CO-2 (blue), and on digestate of encapsulated (red) or non-encapsulated (brown) CO-2 supplements. Peak assignment is provided in Table 2.
